# A National Tribute Fund

**Published:** 1918-06-01

**Authors:** 


ARTHUR STANLEY.

### NURSING AFFAIRS IN IRELAND.

### A National Tribute Fund.

AN influentially attended meeting was held at the Royal Hospital, Dublin, on May 21, to inaugurate a special Irish Fund, as a part of the greater Fund, for the benefit of nurses. This effort, due altogether to the patriotic impulse of Sir Arthur Stanley, promises well. Sir John Arnott, Chairman of the *Irish Times,* and Lady Arnott, D.B.E., one of Dublin's most ardent war workers, have taken the matter in hand with enthusiasm. If their co-operation in connection with the Our Day Fund for the Red Cross is any index to what may be achieved for nurses, a substantial measure of success is assured. Owing to the help afforded by the *Irish Times* the striking sum of £62,000 was collected by the three southern provinces.

The Marchioness of Waterford presided and made a stirring appeal. Taking account of the various factions which unfortunately prevail in nursing, as in other movements here, Lady Waterford is to be congratulated on the adroit manner in which she avoided any possibility of giving offence. "The original objects of the collection," she said, "were to endow the College of Nursing, and to provide a tribute for the individual nurses. Here in Ireland we are advised to concentrate our efforts on a collection for the latter object—namely, the Tribute Fund, which, as its name implies, is a tribute from the nation to the nurses, a perpetual testimony to their magnificent work in the military Services, and also in safeguarding the health of the nation. . . . The Fund will be for certificated nurses, and the money collected in Ireland will be earmarked for Irish nurses. How the Fund will be administered is not yet decided, but we may trust Sir Arthur Stanley to see that it is placed in impartial and wise keeping."

This is a sufficiently broad basis, and ought to have the effect of putting Ireland on her honour. The earmarking idea may sound insular, if not selfish, and it may also have the effect of limiting the benefit Ireland may derive from a great national movement to the amount of the Irish contribution. But, on the other hand, if this condition were omitted, a large number of subscriptions would be withheld. The element of suspicion which pervades the atmosphere has always to be taken into account. Even when the Our Day Fund was being collected many wealthy and loyal subscribers expressed a wish to earmark their contributions for Ireland, unmindful of the obvious fact that the tents of the Red Cross armies are pitched behind the battle front.

Mr. Justice Ross supported Lady Waterford's appeal with that whole-hearted enthusiasm that might be expected from one of Ireland's most patriotic citizens. Paying due tribute to the courage and devotion of auxiliary nurses sent out by the Order of St. John and the British Red Cross Society, Judge Ross said that the trained nurses were the nucleus of them all. They had never failed in their hour of trial, and the people owed them a debt which they could never repay. Judge Ross went on to point out that "there was a proposition which was not before the Irish people about a College to be established [*sic*] in London, but they in Ireland were taking up another position, which they thought would be more acceptable and more capable of gaining support, and that was to raise a kind of hostel or place of support for trained nurses when they were no longer able for their work."

Lady Arnott has consented to act as Chairman of the Executive Committee. The organising and collecting is in the hands of Mr. Henry McLaughlin, who proposes to work through the agency of the wives of His Majesty's Lieutenants in their various counties, and who promise to hand Sir Arthur Stanley £10,000. Miss Macnie and Miss McDonnell are the Honorary Secretaries.

**It should be noted that the "Irish Nurses' Association," or its alter ego "The Irish Nursing Board" was not represented. Apologies for non-attendance from the leaders were read.**

IERNE.

